# Degrade to stay healthy—Proteolytic interplay during inflammation

**DOI:** 10.1371/journal.pbio.3002548

**Published:** 2024-03-07

**Authors:** Christian Münz

**Affiliations:** Viral Immunobiology, Institute of Experimental Immunology, University of Zürich, Zürich, Switzerland

## Abstract

Proteasomes and autophagy constitute the two main proteolytic machineries for cytoplasmic content. This Primer explores a new study in PLOS Biology which demonstrates that autophagy stimulation alters proteasome composition, degrading hyperactive immunoproteasomes and thereby limiting inflammation.

## Teaser

Proteasomes and autophagy constitute the 2 main proteolytic machineries for cytoplasmic content. A new study in *PLOS Biology* now demonstrates that autophagy stimulation alters proteasome composition, degrading hyperactive immunoproteasomes and thereby limiting inflammation.

Cytoplasmic proteins are either unfolded and degraded by proteasomes, or aggregated and delivered via autophagy for lysosomal degradation. These 2 degradation machineries are interconnected because ubiquitination marks substrates for both catabolic pathways [1].

**Fig 1:**
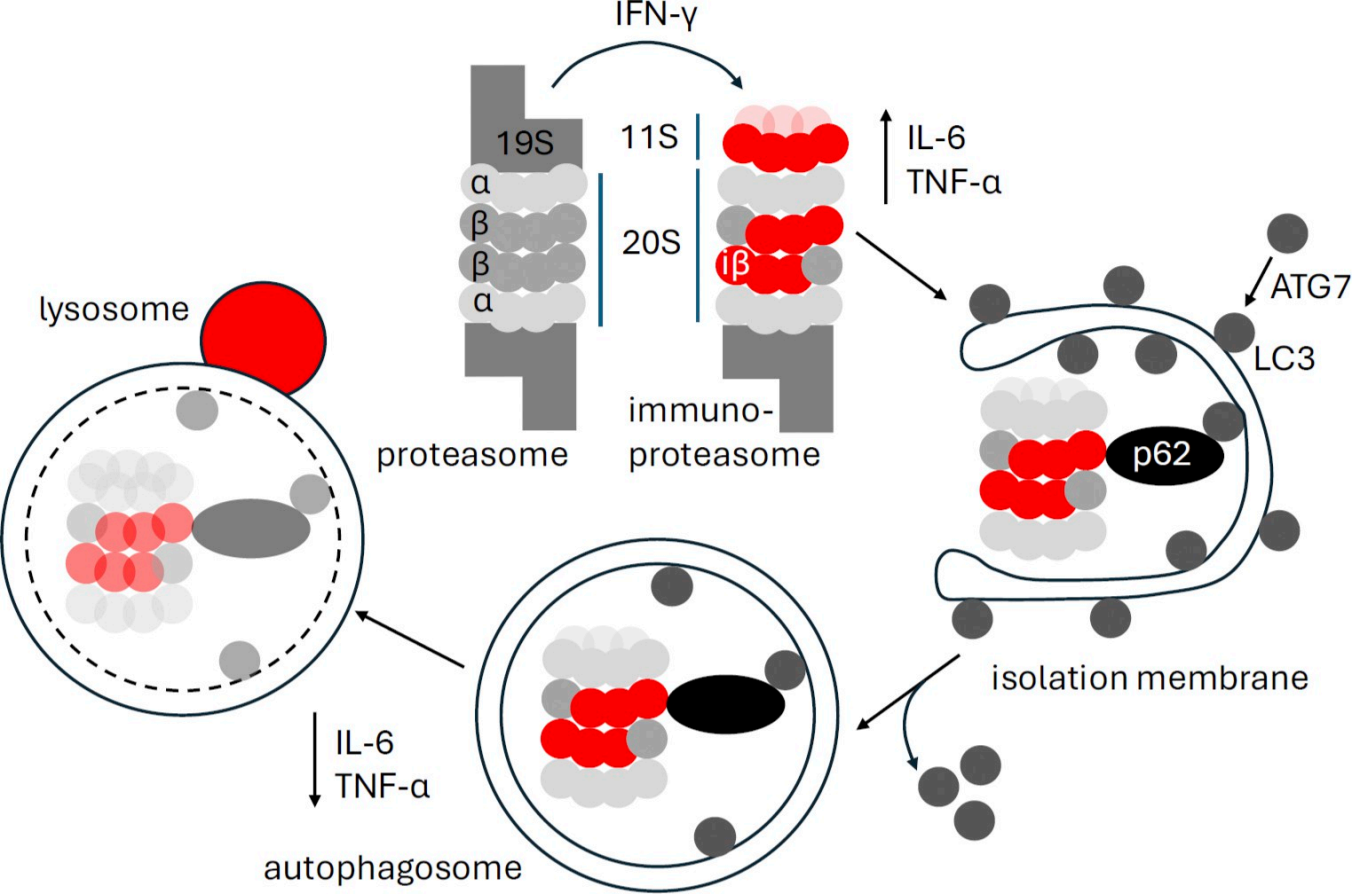
Stimulation of immunoproteasome degradation by macroautophagy ameliorates inflammation. Immunoproteasomes carry inducible catalytic β subunits (iβ1: LMP2; iβ2: MECL-1; iβ: LMP7) and the 11S regulatory particle (PA28). These get induced by, for example, IFN-γ. Immunoproteasomes support inflammation, for example, IL-6 and TNF-α production. Macroautophagy induction leads to the p62 mediated incorporation of immunoproteasomes into autophagosomes. Immunoproteasome degradation after fusion with lysosomes down-regulates inflammation, for example, IL-6 and TNF-α production.

Proteasomes are barrel-like multicatalytic proteases in the cytosol that are composed of α and β subunit rings [2]. Within their inner proteolytic chamber, they contain 6 proteolytic sites (3 per β subunit ring) of 2 β1, 2 β2, and 2 β5 proteolytic activities that are caspase-like, trypsin-like, and chymotrypsin-like. Access to the proteolytic chamber that is composed of the β subunit heptameric rings is guided by the non-catalytic α subunit heptameric rings and adaptors on either end of the 20S proteasome barrel, such as the 19S regulatory particle which admits poly-ubiquitinated proteins to the proteolytic chamber of the proteasome. Upon immune activation and in particular interferon-γ (IFN-γ) stimulation the catalytic β subunits can be replaced by inducible counterparts (LMP2, MECL-1, and LMP7), and an 11S regulatory particle (PA28) can dock to the α subunit rings (**Fig 1**). These modifications increase the processivity of proteasomes, up-regulating their output of octameric or nonameric peptides after cytosolic protein degradation. These modified proteasomes are also called immunoproteasomes because their increased peptide output seems to facilitate presentation of certain peptides on major histocompatibility complex (MHC) class I molecules to cytotoxic CD8^+^ T cells during immune responses.

While inflammatory stimuli, such as IFN-γ, increase immunoproteasome formation, the study by Zhou and colleagues in this *PLOS Biology* issue suggests that macroautophagy selectively degrades them [3]. The authors employed pharmacological macroautophagy induction with the fibroblast growth factor receptor (FGFR) inhibitor LY2874455 and observed selective degradation of immunoproteasomes. During macroautophagy, more than 40 autophagy-related gene products (ATGs) coordinate the degradation of cytoplasmic constituents in lysosomes. At the heart of this process lies the covalent linkage of ubiquitin-like molecules of the LC3 or GABARAP families to primarily phosphatidylethanolamine of newly assembling isolation membranes that then close into a double membrane surrounded autophagosome. These membrane-coupled LC3 and GABARAP proteins are used by autophagy receptors, such as p62, to recruit substrates into the emerging autophagosomes, via cross-linking, for example, poly-ubiquitinated protein aggregates with LC3. After fusion of autophagosomes with lysosomes, this cargo and the inner autophagosomal membrane are degraded [4]. Proteasomes are quite prominent among autophagosome cargo and macroautophagy stimulation reduces proteasome activity in cells [1]. However, some of the proteasomes that are found in autophagosomes might still be functional and contribute to the proteolytic activity of amphisomes that are generated by fusion of autophagosomes with endosomes [5]. Zhou and colleagues demonstrate that p62 is involved in the selective recruitment of immunoproteasomes or at least the inducible β subunits of immunoproteasomes into autophagosomes (**Fig 1**), and that their degradation is ATG7 dependent [3]. These findings suggest that macroautophagy regulates the main cytosolic protease activity, namely proteasomes, and even its catalytic processivity, targeting preferentially immunoproteasomes for degradation.

Which functions of immunoproteasomes are affected by this macroautophagy regulation? Zhou and colleagues emphasize the role of immunoproteasomes during inflammation [3]. Indeed, it had been previously shown that immunoproteasome inhibition ameliorates inflammatory diseases and decreases primarily IL-6, TNF-α, and IL-1β production by myeloid cells [6]. These cytokines or their inactive precursor are transcribed in a nuclear factor kappa B (NF-κB)-dependent fashion [7,8]. While some groups have suggested that the inhibitor of NF-κB (IκB) is degraded by immunoproteasomes more efficiently and thereby leads to increased NF-κB activation [8], others have not observed differences in NF-κB-dependent pro-inflammatory cytokine production in cells that lack individual inducible subunits of immunoproteasomes [7]. However consistent with NF-κB activation by immunoproteasomes, Zhou and colleagues find decreased IL-6 and TNF-α production by a lipopolysaccharide (LPS) stimulated mouse macrophage cell line after pharmacological immunoproteasome inhibition and LY2874455 mediated macroautophagy stimulation (**Fig 1**) [3]. Moreover, LY2874455 stimulation attenuated LPS-induced lung inflammation and dextran sodium sulphate (DSS)-induced colitis. Thus, immunoproteasome degradation seems to be a mechanism by which macroautophagy attenuates inflammation, in addition to degradation of inflammasome substrates and of cytosolic danger and pathogen-associated molecular pattern receptors [9].

Beyond their regulation of inflammation, immunoproteasomes have been initially characterized for their ability to efficiently produce MHC class I ligands for CD8^+^ T cell stimulation. Along these lines proteolytically active proteasomes have been observed in endosomal compartments and seem to facilitate generation of peptides for efficient loading of MHC class I molecules at these sites [5]. Such endosomal MHC class I loading, in contrast to classical MHC class I loading in the endoplasmic reticulum, is especially important for cross-presentation of extracellular antigens to CD8^+^ T cells by dendritic cells during the initiation of immune responses [10]. Such cross-presentation would be particularly efficient if primarily immunoproteasomes would be imported into endosomal compartments via macroautophagy. Therefore, it would be interesting to characterize if p62 dependent immunoproteasome incorporation into autophagosomes allows them to contribute to proteolytic activity in amphisomes, if this import extends to the 11S regulatory particle (PA28), and if this mechanism supports antigen cross-presentation by dendritic cells. Therefore, the macroautophagic import of immunoproteasomes into the endolysosomal system, as described by Zhou and colleagues in this *PLOS Biology* issue, opens up possibilities to target this mechanism to ameliorate inflammatory diseases as well as to possibly increase cross-presentation of extracellular antigens on MHC class I molecules for more efficient CD8^+^ T cell responses.
